# Fatigue and related factors among hotel workers: the effects of emotional labor and non-standard working hours

**DOI:** 10.1186/s40557-014-0051-y

**Published:** 2014-12-18

**Authors:** Ju Jong Lee, Hyun Jey Moon, Kyung-Jae Lee, Joo Ja Kim

**Affiliations:** Department of Occupational & Environment Medicine, Soonchunhyang University Hospital, Seoul, Korea

**Keywords:** Fatigue, Emotional labor, Hotel workers

## Abstract

**Objectives:**

This study assessed fatigue and its association with emotional labor and non-standard working hours among hotel workers.

**Methods:**

A structured self-administered questionnaire was distributed to 1,320 employees of five hotels located in Seoul. The questionnaire survey included questions concerning the participants’ sociodemographics, health-related behaviors, job-related factors, emotional labor, and fatigue. Fatigue was assessed using the Multidimensional Fatigue Scale (MFS). Multiple logistic regression modeling was used to determine the associations between fatigue and emotional labor.

**Results:**

Among male workers, there was a significant association between fatigue and both emotional disharmony (OR=5.52, 95% CI=2.35-12.97) and emotional effort (OR=3.48, 95% CI=1.54-7.86). These same associations were seen among the female workers (emotional disharmony: OR=6.91, 95% CI=2.93-16.33; emotional effort: OR=2.28, 95% CI=1.00-5.16).

**Conclusion:**

These results indicate that fatigue is associated with emotional labor and, especially, emotional disharmony among hotel workers. Therefore, emotional disharmony management would prove helpful for the prevention of fatigue.

## Introduction

The growth of the service industry is characteristic of the industrial structure of a postmodern society. The Korean service industry is experiencing a rapid growth not observed in other industry sectors. According to the statistics bureau’s second quarterly 2011 report, the service sector accounted for 64% of the nation’s Gross Domestic Product (GDP) [1] and 70% of all employment [2], and its growth is expected to increase. Within the service industry, the hotel business is particularly labor intensive. As such, human resources management is critically important for its success. According to the Ministry of Labor Statistics, however, occupational disasters in the food services and lodging industry have increased during the last five years. There were 190 cases in 2006, 220 in 2007, 239 in 2008, 280 in 2009, and 272 in 2010. In 2010, the food services and lodging industry accounted for 7% of overall employment, yet its share of occupational injuries and deaths were 21% and 18%, respectively. This fact demonstrates that those employed by the industry are exposed to a higher rate of occupational hazards than are employees of other industries in the services sector [3]. The data consulted here may not be applicable across the field, since they primarily concern the food services and lodging industry. Nevertheless, a very similar trend is expected for the hotel industry, considering that food service and lodging are at the core of the business.

Hotel operation consists of various aspects, including rooms, food and beverage, back office, kitchen, grounds, and housekeeping. Therefore, it is necessary to take into account various potential occupational hazards [4]. The success of guest services, as well as the food and beverage department, hinges on its employees’ full attentiveness and expressed hospitality, as the business revolves around constant face-to-face interaction with customers in an effort to satisfy their assorted demands [5]. These employees are frequently required to stand or sit still, or to otherwise maintain an awkward and unnatural posture for prolonged periods of time. In the case of the kitchen, the small quarters and extreme indoor temperatures are frequent causes of burns, cuts, and falls [6]. Employees working in the housekeeping and other departments also regularly shoulder physical burden related to repeated use, forceful exertion, and awkward and unnatural postures [7,8]. Hotels operate 24/7, year-round; thus, shift work is indispensable. According to the 2011 survey on working hours conducted by the Ministry of Labor [9], the industry’s average shift work rate was estimated at 15.2%, whereas a figure of 34.0% was estimated for the food services and lodging sectors. As briefly examined here, a wide range of occupational hazards threatening hotel employees’ health and well-being clearly exists. Therefore, continuous research is needed into this issue.

From an occupational health perspective, fatigue is defined as a state of exhaustion resulting from excessive physical and mental labor. Fatigue, in and of itself, is a reversible physiological change, not a disease. However, fatigue may also be viewed as a health alarm, since excessive fatigue can disrupt one’s homeostasis and lead to illnesses [10,11]. Chronic fatigue is difficult to shake off and can accompany other physical and psychological illnesses [12]. Furthermore, some studies have reported that chronic stress may be a predictor of permanent disability [13]. Aside from its clinical effects, fatigue is also known to have sizeable economic and social effects on its sufferers [12]. Chronic fatigue also incurs social costs by disrupting the normal rhythms of daily life, which may lead to work interruption [13,14], lowered productivity, and occupational disasters [15,16]. For these reasons, research in this area has significant implications in terms of workers’ health and productivity.

Fatigue can be distinguished into three different types according to one’s own evaluation: psychological, physical, and neurological [17]. Psychological fatigue refers to central nervous system fatigue and is experienced when one is engaged in a task that requires full attention. Physical fatigue generally refers to muscle fatigue due to physical labor. Finally, neurological fatigue refers to neurological disturbances due to physical and mental labor. In the case of hotel employees, emotional labor, including frequent personal interactions with guests contributes to psychological fatigue, and prolonged periods of maintaining awkward and unnatural posture contributes to physical fatigue. Almost every position available in a hotel accompanies a mix of physical and psychological fatigue risks, although their proportions may vary.

Work-related fatigue occurs when the physical work environment interacts with various other elements, such as personal life circumstances and work-related psychology [18,19]. In the case of the hotel business, since the customer-first principle and customer satisfaction are hailed as the number one priority, the emotion labor demanded of employees is intense, and this can trigger psychological exhaustion and extreme fatigue. In 1983, Hochschild [20] first conceptualized emotional labor as the “regulation of one’s emotion to accommodate the feelings of others.” Emotional labor, in general, can be defined as effort, planning, and the management process involved in conducting an interpersonal task within the workplace, according to organizational expectations [21]. Emotional labor is drawing increased global attention in the postmodern world as the demand for it grows rapidly alongside with the continuously expanding service industry [22]. As for effects of emotional labor, a sense of achievement and self-efficacy [23] are occasionally mentioned, while the act of smiling reportedly triggers positive emotions in a person by increasing circulation and stimulating certain neurotransmitters [24]. Unfortunately, however, the industry’s negative consequences, such as elevated stress levels [25], burnout [26,27], depression [28], somatic symptoms [29], and musculoskeletal symptoms [30] are assessed as being more pronounced than the positive ones, particularly as the degree of emotional labor required of a person increases. Certainly, there is a need for investigating the various health effects of emotional labor, but related studies are currently lacking.

As such, examining the association between hotel employees’ emotional labor and fatigue seemed to be a reasonable place to begin, as the latter is considered to be an initial health alarm. The present study aims to identify and examine the factors associated with the fatigue experienced by hotel employees as a result of emotional labor.

## Materials and methods

### Participants

From June 1 to October 21, 2011, a survey was conducted on 1,320 hotel employees working at 5 Seoul hotels. From these, 1,072 voluntary survey responses were collected (response rate: 81.2%). We eliminated 56 incomplete responses, resulting in 1,016 survey responses.

### Written survey

Self-administered questionnaires contained questions designed to investigate general and occupational characteristics, as well as the level of fatigue and emotional labor perceived by the employees. Questions on alcohol use, tobacco use, and exercise habits offered response choices of yes or no, while a question on nightly sleep duration offered structured responses of “more than 6 hours” and “less than 6 hours.” Sleep satisfaction was surveyed through the following question: “The amount of nightly sleep I get is adequate for recovering from fatigue.” Those who responded with “more than adequate” or “adequate” were put into the sleep-satisfied group, while those who responded with “not adequate” or “very inadequate” were classified as sleep-dissatisfied. Body mass index (BMI) was calculated by measuring weight (kilogram) and height (meter). According to the World Health Organization’s (WHO) Asia-Pacific Standards [31] participants were considered to be “obese” if their BMI was over 25 kg/m^2^. Marital status was distinguished into categories: single (including divorced, separated, and widowed) and married.

As mentioned above, hotel operation can be largely divided into the division of rooms and the administration of the food and beverage department. Rooms division is subsequently divided into guest services (front desk, doors, bell desk, etc.) and housekeeping, while food and beverage service is divided into kitchen and dining hall (waitstaff). Management can be divided into back office (finance, sales and promotion) and grounds (maintenance and repair, electricity, etc.) Based on this, we selected the following categories for our study: guest services, dining hall, kitchen, housekeeping, back office, and grounds. Weekly working hours were divided into the following categories based on the 40-hour workweek as defined in the labor law: less than 40 hours, 40–59 hours, and more than 60 hours. Shift workers were defined as those who responded in the affirmative to the question, “Are you currently working shifts?” Overtime workers were defined as those who answered in the affirmative to the question “Do you work overtime?” To the question,”What is the intensity level of your tasks, in general?” those who responded “not very intense” or “moderate” were classified into the low work intensity group, while those who responded “fairly intense” or “very intense” were classified into the high work intensity group.

1. Fatigue level
  A Korean version of the MFS, which is composed of 19 items, was used to measure fatigue levels. The scale was developed by Chang [32] based on Schwartz et al.’s Fatigue Assessment Inventory (FAI), and the validity and reliability of the instrument were proven through usefulness tests. The MFS is designed to evaluate an individual’s fatigue level as experienced over the previous 2 weeks, and it consists of 8 questions on global fatigue, 6 questions on daily dysfunctioning, and 5 questions on situational fatigue. For each question, the respondents are expected to score their fatigue level on a 7-point Likert scale. The scores are then added up to determine the fatigue level, with a higher score indicating a higher level of fatigue. For the purpose of comparison, the data sets were divided into quartiles: the highest quartile (Q4), second highest quartile (Q3), second lowest quartile (Q2), and lowest quartile (Q1) Subsequent analyses were performed with Q4 defined as the “fatigued group” and Q1, Q2, and Q3 together defined as the “normal group. Cronbach’s α value was 0.941 in an MFS reliability test.
2. Intensity level of emotional labor
  To measure this construct, we used Ahn et al.’s survey [33], which was developed by adapting Morris et al.’s emotional labor survey [21]. The survey contained a total of 9 questions on a 5-point Likert scale, which were distributed between two subcategories of emotional effort and emotional dissonance. “Emotional effort” refers to one’s effort to suppress and conceal his/her authentic emotions from customers, while “emotional dissonance” refers to the pain one perceives due to the discrepancy between his/her authentic emotions and the emotions he/she is required to display according to the organization’s rules. In order to clearly identify the association between emotional labor and fatigue, quartiles were used to evaluate emotional effort and emotional dissonance. Cronbach’s α measured 0.735 (4 items) for emotional effort and 0.823 (5 items) for emotional dissonance.

### Data analysis

First, a chi-square test was performed to analyze the association between general and occupational characteristics and perceived fatigue level. Considering the relatively high proportion of female employees in the hotel industry, as well as the distinct gender differences present, the analysis was gender-stratified.

Second, a logistic regression analysis was performed with fatigue level as the dependent variable and the variables that appeared to be significantly associated with fatigue level in the chi-square test as the independent variables. Univariate logistic regression analysis was initially performed, followed by a multiple logistic regression analysis for Model I and Model II. Statistical analysis was performed using the SPSS14.0 program with a significance level set at p < 0.05.

## Results

### General and occupational characteristics and gender distribution

Of the 1,016 survey respondents, 620 (61.0%) were male and 396 (39.0%) were female. The median age was 37.2 years (39.3 for males, and 33.9 for females). Those in their twenties made up the majority, followed by those in their thirties, forties, and fifties. In terms of job type, 30.7% was employed in the dining hall department, followed by kitchen (23.2%), guest services (19.3%), back office (13.7%), grounds (7.9%), and housekeeping (5.2%.) As for weekly working hours, the majority of participants worked 40–59 hours at 59.7%, followed by those who worked less than 40 hours (32.9%) and those who worked more than 60 hours (7.4%). Those who had worked for less than 10 years accounted for the majority, at 48.0%, followed by those who had been working 10–19 years (29%), and those who had worked more than 20 years (23.0%). Shift work accounted for 57.8%, and overtime accounted for 50.6%.

General gender differences were observed as follows. In terms of age, males in their thirties and females in their twenties accounted for the majority of the participants. As for marital status, more males were married than females (p < 0.001). In terms of tobacco use, alcohol use, regular exercise, and obesity, significantly more males responded in the affirmative on all counts (p < 0.001). Additionally, more males responded affirmatively to sleep satisfaction (p < 0.001), but no significant differences were observed between the genders in terms of actual hours of sleep.

As for occupational characteristics, the majority of male employees worked in the kitchen, followed by the dining hall, whereas the majority of female employees worked in the dining hall, followed by the guest services (p < 0.001). In terms of work tenure, male employees were fairly evenly distributed across all groups, whereas female employees were visibly concentrated in the “less than 10 years” group, at 61.6% (p < 0.001).

In terms of weekly working hours, more male employees worked “more than 60 hours,” and the men worked more overtime (p < 0.05). As for emotional labor, female employees’ concentration in the highest quartile (Q4) was pronounced for both emotional effort (p < 0.001) and emotional dissonance (p < 0.05) compared with the male employees. However, gender differences in shift work and work intensity were not found to be statistically significant (Table 1).

**Table 1:** **Participants’ general and work-related characteristics**

**Variables**	**Total (N = 1,016)**	**Male (N = 620)**	**Female (N = 396)**
	**N (%)**	**N (%)**	**N (%)**
Age(years)*			
20-29	328 (32.3)	142 (22.9)	186 (47.0)
30-39	310 (30.5)	197 (31.8)	113 (28.5)
40-49	195 (19.2)	153 (24.7)	42 (10.6)
≥50	183 (18.0)	128 (20.6)	55 (13.9)
Marital status*			
Married	524 (51.6)	373 (60.2)	151 (38.1)
Single	492 (48.4)	247 (39.8)	245 (61.9)
Smoking*			
No	680 (66.9)	301 (48.5)	379 (95.7)
Yes	336 (33.1)	319 (51.5)	17 (4.3)
Alcohol drinking*			
No	229 (22.5)	92 (14.8)	259 (34.6)
Yes	787 (77.5)	528 (85.2)	137 (65.4)
Regular exercise*			
No	282 (27.8)	138 (22.3)	144 (36.4)
Yes	734 (72.2)	482 (77.7)	252 (63.6)
Daily sleeping hours			
≤6	516 (50.8)	309 (49.8)	207 (52.3)
>6	500 (49.2)	311 (50.2)	189 (47.7)
Sleep satisfaction*			
Satisfied	533 (52.5)	363 (58.5)	170 (42.9)
Not satisfied	483 (47.5)	257 (41.5)	226 (57.1)
BMI(kg/m^2^)*			
<25	781 (76.9)	422 (68.1)	359 (90.7)
≥25	235 (23.1)	198 (31.9)	37 (9.3)
Department*			
Guest services	196 (19.3)	97 (15.6)	99 (25.0)
Housekeeping	53 (5.2)	25 (4.0)	28 (7.1)
Dining hall	312 (30.7)	157 (25.3)	155 (39.1)
Kitchen	236 (23.2)	201 (32.4)	35 (8.8)
Back office	139 (13.7)	62 (10.0)	77 (19.4)
Grounds	80 (7.9)	78 (12.6)	2 (0.5)
Work tenure(years)*			
≤9	487 (48.0)	243 (39.2)	244 (61.6)
10-19	295 (29.0)	189 (30.5)	106 (26.8)
≥20	234 (23.0)	188 (30.3)	46 (11.6)
Weekly working hours^†^			
≤39	334 (32.9)	216 (34.8)	118 (29.8)
40-59	607 (59.7)	348 (56.1)	259 (65.4)
≥60	75 (7.4)	56 (9.0)	19 (4.8)
Shift work			
No	429 (42.2)	268 (43.2)	161 (40.7)
Yes	587 (57.8)	352 (56.8)	235 (59.3)
Overtime work^†^			
No	502 (49.4)	291 (46.9)	211 (53.3)
Yes	514 (50.6)	329 (53.1)	185 (44.7)
Work intensity			
Low	595 (58.6)	376 (60.6)	219 (55.3)
High	421 (41.4)	244 (39.4)	177 (44.7)
Emotional effort*			
Low (Q1)	298 (29.3)	200 (32.3)	98 (24.7)
Middle (Q2)	256 (25.2)	161 (26.0)	95 (24.0)
High (Q3)	253 (24.9)	157 (25.3)	96 (24.2)
Very high (Q4)	209 (20.6)	102 (16.5)	107 (27.0)
Emotional disharmony^†^			
Low (Q1)	235 (23.1)	150 (24.2)	85 (37.0)
Middle (Q2)	274 (27.0)	161 (26.0)	113 (28.5)
High (Q3)	240 (23.6)	162 (26.1)	78 (19.7)
Very high (Q4)	267 (26.3)	147 (23.7)	120 (30.3)

*p < 0.001, ^†^p < 0.05 by chi-square test.

Q1, Q2, Q3, and Q4 indicate 1st quartile, 2nd quartile, 3rd quartile, and 4th quartile.

### Analysis of gender differences in fatigue distribution according to general and occupational characteristics

In terms of general characteristics, male employees were typically in their thirties (p < 0.05), did not exercise (p < 0.05), were not satisfied with the amount of sleep they got (p < 0.001), and were found to experience higher levels of fatigue. Female employees were typically in their twenties (p < 0.05), did not exercise (p < 0.05), were not satisfied with the amount of sleep they got (p < 0.001), and were found to experience higher levels of fatigue.

In relation to occupational characteristics, male employees who worked in the kitchen (p < 0.05), had an average work tenure of 10–19 years (p < 0.05), worked more than 60 hours per week (p < 0.05), including overtime (p < 0.05) and shift work (p < 0.05) felt more fatigue. These men also experienced greater work intensity (p < 0.001) and belonged to the highest quartiles for emotional effort (p < 0.001) and emotional dissonance (p < 0.001). As for female employees, those who had a work tenure of less than 10 years (p < 0.05), worked more than 60 hours per week (p < 0.05), including overtime (p < 0.05), who had higher work intensity (p < 0.05), and who belonged to the highest quartiles for emotional effort (p < 0.001) and emotional dissonance (p < 0.001) were found to experience a higher level of fatigue (Table 2).

**Table 2:** Relationships of general and work-related characteristics to fatigue* by gender

**Variables**	**Male (N = 620)**		**Female (N = 396)**	
	**Normal**	**High**	**Normal**	**High**
Age(years)				
20–29	121 (23.5)	21 (20.0)‡	119 (45.6)	67 (49.6)^‡^
30–39	152 (29.5)	45 (42.9)	65 (24.9)	48 (35.6)
40–49	128 (24.9)	25 (23.8)	28 (10.7)	14 (10.4)
≥50	114 (22.1)	14 (13.3)	49 (18.8)	6 (4.4)
Marital status				
Married	304 (59.0)	69 (65.7)	106 (40.6)	45 (33.3)
Single	211 (41.0)	36 (34.3)	155 (59.4)	90 (66.7)
Smoking				
No	244 (47.4)	57 (54.3)	250 (95.8)	129 (95.6)
Yes	271 (52.6)	48 (45.7)	11 (4.2)	6 (4.4)
Alcohol drinking				
No	75 (14.6)	17 (16.2)	97 (37.2)	40 (29.6)
Yes	440 (85.4)	88 (83.8)	164 (62.8)	95 (70.4)
Regular exercise				
No	104 (20.2)	34 (32.4)‡	82 (31.4)	62 (45.9)^‡^
Yes	411 (79.8)	71 (67.6)	179 (68.6)	73 (54.1)
Daily sleeping hours				
≤6	256 (49.7)	53 (50.5)	137 (52.5)	70 (51.9)
>6	259 (50.3)	52 (49.5)	124 (47.5)	65 (48.1)
Sleep satisfaction				
Satisfied	318 (61.7)	45 (57.1)^†^	130 (49.8)	40 (29.6)^†^
Not satisfied	197 (38.3)	60 (42.9)	131 (50.2)	95 (70.4)
BMI(kg/m^2^)				
<25	354 (68.7)	68 (64.8)	231 (88.5)	128 (94.8)^‡^
≥25	161 (31.3)	37 (35.2)	30 (11.5)	7 (5.2)
Department				
Dining hall	127 (24.7)	30 (28.6)‡	96 (36.8)	59 (43.7)
Guest services	82 (15.9)	15 (14.3)	65 (24.9)	34 (25.2)
Housekeeping	22 (4.3)	3 (2.9)	23 (8.8)	5 (3.7)
Kitchen	154 (29.9)	47 (44.8)	24 (9.2)	11 (8.1)
Back office	56 (10.9)	6 (5.7)	51 (19.5)	26 (19.3)
Grounds	74 (14.4)	4 (3.8)	2 (0.8)	0 (0.0)
Work tenure(years)				
≤9	202 (39.2)	41 (39.0)‡	152 (58.2)	92 (68.1)^‡^
10–19	148 (28.7)	41 (39.0)	70 (26.8)	36 (26.7)
≥20	165 (32.0)	23 (22.0)	39 (14.9)	7 (5.2)
Weekly working hours				
≤39	191 (37.1)	25 (23.8)‡	88 (33.7)	30 (22.2)^‡^
40–59	285 (55.3)	63 (60.0)	164 (62.8)	95 (70.4)
≥60	39 (7.6)	17 (16.2)	9 (3.4)	10 (7.4)
Shift work				
No	234 (45.4)	34 (32.4)‡	121 (46.4)	40 (29.6)^‡^
Yes	281 (54.6)	71 (67.6)	140 (53.6)	95 (70.4)
Overtime work				
No	257 (49.9)	34 (32.4)‡	153 (58.6)	58 (43.0)^‡^
Yes	258 (50.1)	71 (67.6)	108 (41.4)	77 (57.0)
Work intensity				
Low	331 (64.3)	45 (42.9)^†^	156 (59.8)	63 (46.7)^‡^
High	184 (35.7)	60 (57.1)	105 (40.2)	72 (53.3)
Emotional effort				
Low (Q1)	189 (36.7)	11 (10.5)^†^	84 (32.2)	14 (10.4)^†^
Middle (Q2)	138 (26.8)	23 (21.9)	67 (25.7)	28 (20.7)
High (Q3)	119 (23.1)	38 (36.2)	58 (22.2)	38 (28.1)
Very high (Q4)	69 (13.4)	33 (31.4)	52 (19.9)	55 (40.7)
Emotional disharmony				
Low (Q1)	142 (27.6)	8 (7.6)^†^	76 (29.1)	9 (6.7)^†^
Middle (Q2)	146 (28.3)	15 (14.3)	87 (33.3)	26 (19.3)
High (Q3)	135 (26.2)	27 (25.7)	47 (18.0)	31 (23.0)
Very high (Q4)	92 (17.9)	55 (52.4)	51 (19.5)	69 (51.1)

Unit: N(%).

*fatigue (Multidimensional Fatigue Scale) was dichotomized as either normal (Q1, Q2, Q3) or high (Q4).

^†^p < 0.001, ^‡^p < 0.05 by chi-square test.

Q1, Q2, Q3, and Q4 indicates1st quartile, 2nd quartile, 3rd quartile, and 4th quartile.

### Logistic regression analysis results for fatigue level

In order to examine the factors influencing fatigue level, MFS fatigue scores were placed into a high risk group (Q4) and the normal group (Q1,Q2,Q3) by gender for a binomial logistic regression analysis. All factors that were found to have a significant correlation with fatigue distribution in the cross analysis were used as the independent variables: age, regular exercise, sleep satisfaction status, BMI, department, work tenure, weekly working hours, shift work status, overtime work status, work intensity, emotional effort, and emotional dissonance. Fatigue level was the dependent variable. The variance inflation factor (VIF) of each independent variable was less than 10, and upon verification of no multicollinearity, a logistic regression analysis was performed.

In the case of male employees, the risk of being identified within a high fatigue risk group rose significantly in the Q3 (OR = 3.30, 95% CI = 1.49-7.28) and the Q4 (OR = 3.72, 95% CI = 1.60-8.64) for emotional effort, and in the Q3 (OR = 2.65, 95% CI = 1.11-6.33) and the Q4 (OR = 5.70, 95% CI = 2.36-13.77) for emotional dissonance, compared with the lowest quartile (Q1) (Table 3). risk of being identified in the high fatigue risk group rose significantly in the Q3 (OR = 5.10, 95% CI = 2.09-12.48) and Q4 (OR = 6.59, 95% CI = 2.73-15.92) for emotional dissonance. However, no statistically significant risk was observed in the domain of emotional effort. Also, the risk of being identified in the high fatigue risk group rose significantly for the female employees who engaged in shift work (OR = 1.94, 95% CI = 1.09-3.46),who had 10–19 years of work tenure (OR = 0.42, 95% CI = 0.19-0.93) or over 20 years (OR = 0.20, 95% CI = 0.05-0.75), and who were not satisfied with their sleep durations (OR = 1.71, 95% CI = 1.01-2.90) (Table 4).

**Table 3:** **Logistic regression analysis on fatigue in male hotel workers**

**Variables**	**Unadjusted**		**Adjusted**			
			Model I*		**Model II** ^**†**^	
	**OR**	**(95% CI)**	**OR**	**(95% CI)**	**OR**	**(95% CI)**
Age(years)						
20–29	1.00		1.00		1.00	
30–39	1.71	(0.96-3.02)	1.08	(0.53-2.22)	1.16	(0.55-2.45)
40–49	1.13	(0.60-2.12)	0.90	(0.35-2.30)	1.13	(0.42-3.02)
≥50	0.71	(0.34-1.46)	1.07	(0.36-3.19)	1.57	(0.48-5.12)
Sleep satisfaction						
Satisfied	1.00		1.00		1.00	
Not satisfied	2.15	(1.41-3.29)	1.48	(0.92-2.39)	1.39	(0.83-2.35)
Work tenure (years)						
≤9	1.00		1.00		1.00	
10–19	1.37	(0.84-2.21)	0.97	(0.50-1.88)	0.85	(0.43-1.70)
≥20	0.69	(0.40-1.19)	0.67	(0.27-1.69)	0.56	(0.22-1.45)
Weekly working hours						
≤39	1.00		1.00		1.00	
40–59	1.69	(1.03-2.78)	1.18	(0.68-2.06)	1.08	(0.61-1.91)
≥60	3.33	(1.64-6.75)	2.48	(1.12-5.48)	2.22	(0.98-5.04)
Shift work						
No	1.00		1.00		1.00	
Yes	1.74	(1.12-2.71)	1.59	(0.97-2.60)	1.64	(0.95-2.80)
Overtime work						
No	1.00		1.00		1.00	
Yes	2.08	(1.34-3.24)	1.32	(0.80-2.17)	1.34	(0.79-2.30)
Emotional effort						
Low (Q1)	1.00		1.00		1.00	
Middle (Q2)	2.86	(1.35-6.07)	1.92	(0.87-4.25)	2.06	(0.91-4.65)
High (Q3)	5.49	(2.70-11.15)	3.06	(1.42-6.62)	3.30	(1.49-7.28)
Very high (Q4)	8.22	(3.94-17.15)	3.48	(1.54-7.86)	3.72	(1.60-8.64)
Emotional disharmony						
Low (Q1)	1.00		1.00		1.00	
Middle (Q2)	1.82	(0.75-4.44)	1.19	(0.47-3.02)	1.22	(0.48-3.11)
High (Q3)	3.55	(1.56-8.09)	2.70	(1.15-6.35)	2.65	(1.11-6.33)
Very high (Q4)	10.61	(4.83-23.30)	5.52	(2.35-12.97)	5.70	(2.36-13.77)

*Model I was adjusted for age, sleep satisfaction, work tenure, weekly working hours, shift work, overtime work, emotional effort, and emotional disharmony.

^†^Model II was additionally adjusted for regular exercise, BMI, department, and work intensity.

OR: odds ratio, CI: confidence interval.

**Table 4:** Logistic regression analysis on fatigue in female hotel workers*

**Variables**	**Unadjusted**		**Adjusted**			
			Model I*		**Model II** ^**†**^	
	**OR**	**(95% CI)**	**OR**	**(95% CI)**	**OR**	**(95% CI)**
Age(years)						
20-29	1.00		1.00		1.00	
30-39	1.31	(0.81-2.12)	1.44	(0.68-3.01)	1.40	(0.65-3.04)
40-49	0.89	(0.44-1.80)	1.97	(0.71-5.46)	2.39	(0.77-7.4)
≥50	0.22	(0.09-0.53)	0.94	(0.29-3.07)	1.04	(0.27-4.07)
Sleep satisfaction						
Satisfied	1.00		1.00		1.00	
Not satisfied	2.36	(1.52-3.67)	1.84	(1.10-3.09)	1.71	(1.01-2.90)
Work tenure (years)						
≤9	1.00		1.00		1.00	
10-19	0.85	(0.53-1.37)	0.46	(0.21-0.98)	0.42	(0.19-0.93)
≥20	0.30	(0.12-0.69)	0.27	(0.08-0.93)	0.20	(0.05-0.75)
Weekly working hours						
≤39	1.00		1.00		1.00	
40-59	1.70	(1.05-2.76)	1.33	(0.75-2.36)	1.29	(0.72-2.30)
≥60	3.26	(1.21-8.78)	2.49	(0.78-7.98)	2.26	(0.70-7.30)
Shift work						
No	1.00		1.00		1.00	
Yes	2.05	(1.32-3.20)	1.89	(1.12-3.22)	1.94	(1.09-3.46)
Overtime work						
No	1.00		1.00		1.00	
Yes	2.08	(1.34-3.24)	1.58	(0.95-2.63)	1.64	(0.96-2.82)
Emotional effort						
Low (Q1)	1.00		1.00		1.00	
Middle (Q2)	2.51	(1.22-5.14)	1.50	(0.68-3.33)	1.40	(0.63-3.12)
High (Q3)	3.93	(1.96-7.90)	2.03	(0.89-4.61)	2.11	(0.90-4.93)
Very high (Q4)	6.35	(3.21-12.54)	2.28	(1.00-5.16)	2.32	(0.98-5.45)
Emotional disharmony						
Low (Q1)	1.00		1.00		1.00	
Middle (Q2)	2.52	(1.11-5.72)	1.81	(0.76-4.29)	1.77	(0.74-4.24)
High (Q3)	5.57	(2.44-12.73)	5.23	(2.18-12.54)	5.10	(2.09-12.48)
Very high (Q4)	11.43	(5.24-24.92)	6.91	(2.93-16.33)	6.59	(2.73-15.92)

*Model I was adjusted for age, sleep satisfaction, work tenure, weekly working hours, shift work, overtime work, emotional effort, and emotional disharmony.

^†^Model II was additionally adjusted for regular exercise, BMI, department, and work intensity.

OR: odds ratio, CI: confidence interval.

## Discussion

This study aimed to identify the factors associated with hotel employees’ fatigue by examining general and occupational characteristics that potentially elevate the intensity of emotional labor and fatigue levels. Our results indicate that the male employees who belong in the highest quartile (Q4) for emotional effort were at 3.7 times greater risk (OR = 3.72, 95% CI = 1.60-8.64) of being identified in the high fatigue risk group. Similarly, male employees who belonged in the highest quartile (Q4) for emotional dissonance were at 5.7 times greater risk (OR = 5.70, 95% CI = 2.36-13.77) of being identified in the high fatigue risk group, which was statistically significant.

As for female employees, those who belonged in the highest quartile (Q4) for emotional dissonance were at 6.6 times greater risk (OR = 6.59, 95% CI = 2.73-15.92) of being identified in the high fatigue risk group, and this finding was also statistically significant. Therefore, it is concluded that emotional labor is highly correlated with fatigue, and emotional dissonance, in particular, exerts a greater influence on fatigue than emotional effort. In his study of flight attendants, Hochschild [20] asserted that emotional dissonance has various negative consequences on psychological well-being, while Morris et al. [21] reported that emotional dissonance causes emotional burnout, which in turn decreases job satisfaction. Abraham [34] asserted that job satisfaction and organizational commitment are influenced by emotional dissonance. A Korean study on bank employees [35] found that emotional dissonance is more highly correlated with depression risk than is emotional effort. A study on government workers [30] also reported that emotional dissonance is associated with musculoskeletal symptoms.

Although independent comparison would be difficult due to the variance in measuring tools and study subjects, existing studies, as well as the present study results, strongly indicate that emotional dissonance exerts a greater negative influence than any other factor on employees’ health. However, the high association between emotional effort and fatigue observed among the male employees needs to be verified through further research in order to find out whether the obtained result was affected by the lack of a standardized tool for measuring emotional labor, or if it was influenced by other factors, such as gender and job type. Employees’ internal emotional states cannot be positive at all times. As such, authentic employee emotions will, at times, deviate from what is observed externally. Employees who feel emotional dissonance generally attempt to change their internal state of mind, rather than reveal their authentic feelings while under the organization’s control. Repetition of this process leads the employee developing a false sense of self, making emotional regulation difficult. This, in turn, subsequently intensifies the level of emotional labor demanded of him/her [21]. As a result, emotional dissonance contributes to job stress and elevates employees’ fatigue levels.

For the female employees in our study, the fatigue cross ratio for each emotional dissonance level (Q2, Q3, and Q4) was higher than for the male employees, and fatigue level diminished significantly with increased work tenure. Among the general population, female fatigue levels are observed to be approximately 1.2–1.7 times greater than males’ [15,36]. Studies on night workers [37] and nurses found that those with shorter work tenure experienced a higher level of fatigue, a finding that supports our study’s findings. Some attribute these results to female employees’ pronounced emotional expressiveness [38], or to the inability of those with shorter work tenure to effectively respond to work demand, or to a lack of a clearly established work relationship with coworkers and supervisors [39]. However, from the service industry’s perspective, the results may be attributable to the fact that female employees with short work tenure tend to hold a lower level position, which consequently demands greater exposure to emotional labor through face-to-face interaction with guests. Additionally, other studies on service industry workers support this explanation with results that indicated higher intensity of emotional labor in female employees with shorter work tenure [40-42].

For female employees, shift work and sleep satisfaction exhibited significant correlations with fatigue level. Direct comparison is difficult because the national rate of shift work reported for the hospitality industry varies widely, from 8.4% to 47.1%, depending on the survey organizations or sample selection methods. Nevertheless, considering the nature of hotel operation, which has to deal with guests around the clock, rate of shift work for the hotel industry alone is expected to be much higher.

In modern society, where professional careers and service industries continue to occupy a bigger share in the market, the number of rotational shift workers is also on the increase. Employees who work rotational shifts may experience negative health effects, including disruption of normal family life and natural circadian rhythm, in addition to sleep disorders [43,44]. Sleep disorders and chronic sleep deprivation, in particular, cause fatigue, which leads to an increased risk of accident [45,46]. The long working hours of the hospitality industry are currently supported by Section 59 of the Labor Standards Act, as the industry is filed under the exemption sector. According to some studies, long working hours and sleep are negatively correlated, and sleep deprivation due to long working hours has similar effects to sleep deprivation due to rotational shift work, which negatively affects fatigue and health. The combination of long working hours and sleep disorders is reported to cause even more intense fatigue. The hotel business has all the potentials elements for contributing to long working hours, sleep disorders, and rotational shift work. As such, care and attention need to be paid from an occupational medical perspective.

Previous studies confirm several variables that mitigate the negative effects of emotional labor. Studies on hotel employees [33,47] and clinical nurses in general hospitals [48] have reported that the support of supervisors and colleagues, as well as emotional intelligence, mitigates the negative effects of emotional labor. Another study on hotel employees reported that supervisor support mitigates emotional burnout, and employers’ excessive push for the “customer-first” principle exacerbates emotional burnout and alienation [47]. Therefore, strengthening the support of colleagues and supervisors will contribute to preventing occupational diseases through lessened negative health effects, including fatigue. Additionally, professional development offered by employers, such as hospitality education and training, should serve as an opportunity to address employees’ emotional labor, in addition to customer satisfaction. Developmental goals should incorporate equipping the employees with solid tools to effectively respond to problematic situations that arise as part of the hospitality profession, rather than always focusing on the positive aspects of the industry.

The limitations of our study are as follows. First, this is a cross-sectional study; thus, it is not possible to clearly identify the cause and effect relationships between fatigue and influencing factors. Second, the study participants were recruited from 5 hotels located in Seoul; thus, generalizing our findings across the board may be a stretch. Third, due to the lack of a standardized tool to measure emotional labor, a scale used in a different study on hotel employees was adopted for this study. Nevertheless, the reliability of the emotional labor survey used in this study was high. Further, reliability and validity were maximized through factor analysis, which indicated that the questions could be divided into two groups (one with 4 items, and one with 5 items) as in the study by Ahn et al. [33].

Despite the aforementioned limitations, our study was able to confirm the close association between hotel employees’ emotional labor, non-standard work schedules (shift work, etc.), and fatigue. Additional significance can be found in the fact that the findings obtained in our study can be used as valuable foundational data for effectively managing hotel employees’ health. Finally, additional research should be conducted on the association between hotel employees’ emotional labor and various other diseases, such as cardiovascular and digestive diseases.
